# Supplementary material for: Characterization of Streptomyces piniterrae sp. nov. and Identification of the Putative Gene Cluster Encoding the Biosynthesis of Heliquinomycins
Source: Microorganisms. 2020 Mar 31;8(4):495. doi: 10.3390/microorganisms8040495 (PMC7232196; doi:10.3390/microorganisms8040495)

Supporting Information

Characterization of *Streptomyces piniterrae* sp. nov. and Identification of the Putative Gene Cluster Encoding the Biosynthesis of Heliquinomycins

Xiaoxin Zhuang[1†], Zhiyan Wang[2†], Chenghui Peng[1], Can Su[2], Congting Gao[1], Yongjiang Wang[2], Shengxiong Huang[2,*], Chongxi Liu[1,*]

[1]Key Laboratory of Agricultural Microbiology of Heilongjiang Province, Northeast Agricultural University, Harbin 150030, China

[2]State Key Laboratory of Phytochemistry and Plant Resources in West China, Kunming Institute of Botany, Chinese Academy of Sciences, Kunming 650201, China

*Correspondence: Chongxi Liu and Shengxiong Huang

E-mail: xizi-ok@163.com, sxhuang@mail.kib.ac.cn

[†]These authors have contributed equally to this work

Table S1. GenBank accession numbers of the sequnces for the *Streptomyces* sequences used.

Strain	Type strain	Whole genome	*trp*B	*rpo*B	*gyr*B	*atp*D	*rec*A
S. piniterrae	jys28^T	SUMB00000000	–	–	–	–	–
S. lydicus	NBRC 13058^T	CP007699	–	–	–	–	–
S. chattanoogensis	DSM 40002^T	–	FJ406361	FJ406306	FJ406194	FJ406138	FJ406250
S. sioyaensis	NRRL B-5408^T	–	MG881219	MG881217	FJ406188	MG881213	MG881215
S. platensis	JCM 4662^T	–	KT389356	KT389007	KT385036	KT384686	KT385387
S. albulus	NRRL-ISP 5492^T	CP026094	–	–	–	–	–
S. olivaceiscleroticus	NBRC 13484^T	–	JF423964	FJ406332	FJ406220	JF424198	JF424058
S. rimosus	KCTC 1077^T	–	JF423950	JF423997	JF424090	JF424184	JF424044
S. kasugaensis	DSM 40819^T	–	KT389276	KT388927	FJ406226	KT384607	KT385306
S. auratus	NRRL 8097^T	–	KT389144	KJ996206	KT384824	KT384475	KT385172
S. rimosus subsp. *paromomycinus*	DSM 41429^T	–	FJ406377	FJ406322	FJ406210	FJ406154	FJ406266
S. nigrescens	NBRC 12894^T	–	FJ406358	FJ406303	FJ406191	FJ406135	FJ406247
S. angustmyceticus	NRRL B-2347^T	–	HQ244495	HQ244490	HQ244480	HQ244475	HQ244485
S. decoyicus	NRRL 2666^T	–	FJ406382	FJ406327	FJ406215	FJ406159	FJ406271
S. tubercidicus	DSM 40261^T	–	FJ406360	FJ406305	FJ406193	FJ406137	FJ406249
S. hygroscopicus subsp. *glebosus*	NBRC 13786^T	–	FJ406381	FJ406326	FJ406214	FJ406158	FJ406270
S. violens	CGMCC 4.1786 ^{T}	–	FJ406380	FJ406325	FJ406213	FJ406157	FJ406269
S. purpurogeneiscleroticus	JCM 4818^T	–	FJ406383	FJ406328	FJ406216	FJ406160	FJ406272
S. ochraceiscleroticus	NBRC 13483^T	–	FJ406352	FJ406297	FJ406185	FJ406129	FJ406241
S. mashuensis	DSM 40221^T	–	FJ406390	FJ406335	FJ406223	FJ406167	FJ406279
S. albofaciens	JCM 4342^T	–	FJ406371	FJ406316	FJ406204	FJ406148	FJ406260
S. chrestomyceticus	DSM 40545^T	–	FJ406372	FJ406317	FJ406205	FJ406149	FJ406261
S. catenulae	DSM 40258^T	–	FJ406375	FJ406320	FJ406208	FJ406152	FJ406264
S. monomycini	DSM 41801^T	–	FJ406403	FJ406347	FJ406236	FJ406180	FJ406292
S. antimycoticus	NBRC 12839^T	–	FJ406367	FJ406312	FJ406200	FJ406144	FJ406256
S. melanosporofaciens	DSM 40318^T	–	FJ406376	FJ406321	FJ406209	FJ406153	FJ406265
S. castelarensis	DSM 40830^T	–	FJ406394	FJ406338	FJ406227	FJ406171	FJ406283
S. albospinus	NBRC 13846^T	–	FJ406369	FJ406314	FJ406202	FJ406146	FJ406258
S. caniferus	NBRC 15389^T	–	KT389168	KT388818	KT384848	KT384499	KT385196
S. inhibens	NEAU-D10^T	QUAC00000000	–	–	–	–	–

Table S2. MLSA distance values for selected strains in this study.

Strains:1, *S. piniterrae* jys28^T; 2, *S. lydicus* NBRC 13058^T; 3, *S. chattanoogensis* DSM 40002^T; 4, *S. sioyaensis* NRRL B-5408^T; 5, *S. platensis* JCM 4662^T; 6, *S. albulus* NRRL-ISP 5492^T; 7, *S. olivaceiscleroticus* NBRC 13484^T; 8, *S. rimosus* KCTC 1077^T; 9, *S. kasugaensis* DSM 40819^T; 10, *S. auratus* NRRL 8097^T; 11, *S. rimosus* subsp. *paromomycinus* DSM 41429^T; 12, *S. nigrescens* NBRC 12894^T; 13, *S. angustmyceticus* NRRL B-2347^T; 14, *S. decoyicus* NRRL 2666^T; 15, *S. tubercidicus* DSM 40261^T; 16, *S. hygroscopicus* subsp. *glebosus* NBRC 13786^T; 17, *S. violens* CGMCC 4.1786 ^{T}; 18, *S. purpurogeneiscleroticus* JCM 4818^T; 19, *S. ochraceiscleroticus* NBRC 13483^T; 20, *S. mashuensis* DSM 40221^T; 21, *S. albofaciens* JCM 4342^T; 22, *S. chrestomyceticus* DSM 40545^T; 23, *S. catenulae* DSM 40258^T; 24, *S. monomycini* DSM 41801^T; 25, *S. antimycoticus* NBRC 12839^T; 26, *S. melanosporofaciens* DSM 40318^T; 27, *S. castelarensis* DSM 40830^T; 28, *S. albospinus* NBRC 13846^T; 29, *S. caniferus* NBRC 15389^T; 30, *S. inhibens* NEAU-D10^T.

Strain	MLSA (Kimura 2-parameter) distance																											
	1	2	3	4	5	6	7	8	9	10	11	12	13	14	15	16	17	18	19	20	21	22	23	24	25	26	27	28	29
1	-																												
2	0.044	-																											
3	0.057	0.049	-																										
4	0.088	0.078	0.069	-																									
5	0.081	0.059	0.063	0.058	-																								
6	0.076	0.057	0.053	0.078	0.066	-																							
7	0.085	0.088	0.080	0.090	0.090	0.081	-																						
8	0.089	0.095	0.096	0.099	0.095	0.093	0.089	-																					
9	0.061	0.069	0.063	0.090	0.076	0.077	0.084	0.087	-																				
10	0.071	0.062	0.047	0.037	0.036	0.068	0.080	0.096	0.067	-																			
11	0.075	0.071	0.069	0.082	0.075	0.072	0.073	0.052	0.069	0.070	-																		
12	0.080	0.066	0.058	0.056	0.031	0.074	0.091	0.100	0.073	0.034	0.077	-																	
13	0.075	0.062	0.059	0.053	0.044	0.075	0.086	0.092	0.070	0.041	0.073	0.038	-																
14	0.080	0.072	0.062	0.061	0.038	0.078	0.090	0.096	0.071	0.036	0.073	0.024	0.041	-															
15	0.085	0.062	0.066	0.059	0.033	0.070	0.095	0.103	0.080	0.043	0.082	0.039	0.049	0.047	-														
16	0.083	0.061	0.065	0.059	0.008	0.068	0.092	0.097	0.078	0.039	0.078	0.031	0.046	0.039	0.035	-													
17	0.090	0.085	0.082	0.089	0.089	0.080	0.041	0.084	0.082	0.082	0.068	0.092	0.084	0.086	0.096	0.091	-												
18	0.090	0.086	0.082	0.091	0.090	0.079	0.039	0.086	0.083	0.084	0.069	0.091	0.084	0.086	0.096	0.091	0.011	-											
19	0.090	0.088	0.084	0.091	0.093	0.082	0.039	0.086	0.083	0.085	0.071	0.096	0.086	0.088	0.100	0.094	0.014	0.012	-										
20	0.094	0.090	0.100	0.102	0.108	0.094	0.093	0.107	0.101	0.102	0.107	0.110	0.099	0.110	0.117	0.110	0.102	0.103	0.101	-									
21	0.087	0.089	0.085	0.102	0.093	0.088	0.078	0.033	0.073	0.088	0.036	0.091	0.086	0.087	0.102	0.095	0.074	0.073	0.074	0.105	-								
22	0.086	0.084	0.083	0.098	0.087	0.086	0.077	0.039	0.078	0.085	0.031	0.089	0.084	0.085	0.096	0.090	0.078	0.079	0.079	0.105	0.027	-							
23	0.086	0.091	0.079	0.097	0.086	0.089	0.080	0.069	0.080	0.084	0.077	0.086	0.079	0.082	0.095	0.088	0.083	0.083	0.083	0.097	0.063	0.058							
24	0.087	0.086	0.088	0.101	0.091	0.090	0.080	0.045	0.079	0.086	0.039	0.090	0.087	0.085	0.099	0.093	0.082	0.082	0.082	0.108	0.029	0.020	0.060	-					
25	0.113	0.110	0.126	0.127	0.120	0.121	0.120	0.125	0.121	0.121	0.121	0.122	0.121	0.123	0.125	0.122	0.120	0.116	0.121	0.105	0.123	0.120	0.129	0.118	-				
26	0.111	0.110	0.125	0.127	0.119	0.121	0.122	0.124	0.120	0.121	0.120	0.121	0.120	0.123	0.124	0.122	0.121	0.118	0.123	0.106	0.122	0.121	0.129	0.119	0.010	-			
27	0.113	0.110	0.127	0.127	0.120	0.120	0.120	0.124	0.120	0.122	0.120	0.121	0.120	0.123	0.125	0.122	0.120	0.116	0.121	0.104	0.122	0.120	0.128	0.118	0.004	0.009	-		
28	0.055	0.050	0.062	0.081	0.074	0.068	0.084	0.098	0.074	0.066	0.075	0.074	0.072	0.079	0.076	0.075	0.085	0.083	0.085	0.084	0.087	0.087	0.091	0.094	0.115	0.114	0.115	-	
29	0.082	0.075	0.073	0.060	0.046	0.082	0.094	0.095	0.082	0.043	0.075	0.043	0.033	0.041	0.054	0.047	0.090	0.091	0.096	0.108	0.091	0.087	0.090	0.089	0.122	0.121	0.122	0.079	-
30	0.064	0.055	0.045	0.067	0.057	0.063	0.077	0.088	0.056	0.043	0.064	0.051	0.047	0.052	0.065	0.059	0.077	0.077	0.080	0.096	0.078	0.078	0.077	0.081	0.120	0.119	0.120	0.061	0.055

Table S3. Cultural characteristics of strain jys28^T, *S. lydicus* NBRC 13058^T and *S. chattanoogensis* DSM 40002^T.

**Characteristic**	**jys28^T**	***S. lydicus* NBRC 13058^T**	***S. chattanoogensis* DSM**
**Growth on ISP 1:**			
Aerial mycelium	Medium Gray	None	None
Substrate	Moderate Yellowish Brown	Light Yellow	Light Yellow
**Growth on ISP 2:**			
Aerial mycelium	Yellowish White	White	White
Substrate	Dark Yellowish Brown	Brown Brilliant Orange	Dark Orange Yellow
Diffusible pigment	Strong Yellowish Brown	None	None
**Growth on ISP 3:**			
Aerial mycelium	Light Gray	Yellowish Gray	Light Yellowish Green
Substrate	Dark Purplish Red	Light Greenish Yellow	Brilliant Yellowish Green
Diffusible pigment	Dark Purple	None	None
**Growth on ISP 4:**			
Aerial mycelium	Grayish Yellowish Green	White	Light Gray
Substrate	Moderate Reddish Brown	Pale Yellow	Vivid Yellow
Diffusible pigment	None	None	Deep Orange Yellow
**Growth on ISP 5:**			
Aerial mycelium	White	Light Gray	Yellowish White
Substrate	Dark Reddish Brown	Grayish Yellow	Brilliant Greenish Yellow
Diffusible pigment	Dark Reddish Brown	None	Brilliant Orange Yellow
**Growth on ISP 6:**			
Aerial mycelium	Yellowish White	None	None
Substrate	Brilliant Yellow	Light Yellow	Light Yellow
Diffusible pigment	Brilliant Orange Yellow	None	None
**Growth on ISP 7:**			
Aerial mycelium	White	Light Gray	White
Substrate	Dark Reddish Brown	Grayish Reddish Brown	Brilliant Greenish Yellow
Diffusible pigment	Dark Reddish Brown	None	None
**Growth on NA:**			
Aerial mycelium	White	Light Greenish Gray	Yellowish White
Substrate	Dark Reddish Brown	Moderate Greenish	Vivid Greenish Yellow
Diffusible pigment	Dark Reddish Brown	None	Brilliant Orange Yellow
**Growth on CA:**			
Aerial mycelium	Dark Gray	None	White
Substrate	Dark Reddish Brown	Very Pale Green	Pinkish Gray
Diffusible pigment	Dark Reddish Brown	None	None
**Growth on MBA:**			
Aerial mycelium	Yellowish White	None	None
Substrate	Light Greenish Yellow	Pale Greenish Yellow	Pale Greenish Yellow

Table S4. Deduced organization of the heliquinomycins biosynthetic gene cluster in *S. piniterrae* jys28^{T}.

Protein	Proposed Function	Sequence Similarity (Protein, Origin)	Similarity	Accession Number
4024	Transcriptional activator	GrhR1, *S.* sp. JP95	74.1%	AAM33679
4025	Transcriptional activator	GrhR2, *S.* sp. JP95	83.0%	AAM33680
4026	Thioesterase	GrhD, *S.* sp. JP95	72.1%	AAM33656
4027	Acyl carrier protein	GrhC, *S.* sp. JP95	70.1%	AAM33655
4028	Cyclase	GrhE, *S.* sp. JP95	57.9%	AAM33657
4029	Acyl-CoA dehydrogenase	*S.* sp. Ru73	77.3%	WP_103830939
4030	Phosphopantetheinyl transferase	GrhF, *S.* sp. JP95	65.2%	AAM33658
4031	Amino acid adenylation domain-containing protein	entF, *S.* sp. YIM 130001	40.6%	WP_119294030
4032	Unknown	*S.* sp. NRRL S-646	68.1%	WP_051834117
4033	acyl-CoA carboxylase subunit beta	GrhG, *S.* sp. JP95	89.5%	AAM33659
4034	acyl-CoA carboxylase subunit epsilon	GrhH, *S.* sp. JP95	47.7%	AAM33660
4035	Unknown	GrhI, *S.* sp. JP95	91.8%	AAM33661
4036	FAD-binding oxidoreductase	GrhO1, S. sp. JP95	82.4%	AAM33667
4037	GNAT family N-acetyltransferase	GrhJ, *S.* sp. JP95	80.7%	AAM33662
4038	FAD-dependent monooxygenase	GrhO5, *S.* sp. JP95	80.6%	AAM33672
4039	FAD-dependent monooxygenase	GrhO6, *S.* sp. JP95	84.4%	AAM33673
4040	Methyltransferase	GrhL, *S.* sp. JP95	86.7%	AAM33664
4041	FAD-dependent monooxygenase	GrhO8, *S.* sp. JP95	85.4%	AAM33675
4042	FAD-dependent oxidoreductase	GrhO9, *S.* sp. JP95	87.3%	AAM33676
4043	Unknown	GrhM, *S.* sp. JP95	92.7%	AAM33665
4044	Unknown	GrhN, *S.* sp. JP95	69.5%	AAM33666
4045	Class II glutamine amidotransferase	GrhP, *S.* sp. JP95	87.0%	AAM33677
4046	Cyclase	GrhQ, *S.* sp. JP95	90.2%	AAM33678

4047	Cyclase	GrhS, *S.* sp. JP95	83.7%	AAM33682
4048	Ketosynthase alpha	GrhA, *S.* sp. JP95	90.3%	AAM33653
4049	Ketosynthase beta	GrhB, *S.* sp. JP95	85.4%	AAM33654
4050	Bifunctional cyclase/3-oxoacyl-ACP reductase	GrhT, *S.* sp. JP95	89.7%	AAM33685
4051	3-oxoacyl-ACP reductase	GrhO10, *S.* sp. JP95	82.3%	AAM33668
4052	Antibiotic biosynthesis monooxygenase	GrhU, *S.* sp. JP95	79.6%	AAM33683
4053	Antibiotic biosynthesis monooxygenase	GrhV, *S.* sp. JP95	75.0%	AAM33684
4054	Cytochrome P450	RubU, *S. collinus*	65.3%	AAM97370
4055	SAM-dependent methyltransferase	pfam04672, *S. collinus*	61.8%	AAM97375
4056	3-oxoacyl-ACP reductase	GrhO2, *S.* sp. JP95	61.5%	AAM33669
4057	Cytochrome P450	GrhO3, *S.* sp. JP95	36.8%	AAM33670
4058	NADPH:quinone oxidoreductase	GrhO7, *S.* sp. JP95	57.7%	AAM33674
4059	Efflux protein	GrhK, *S.* sp. JP95	47.0%	AAM33663
4060	SDR family oxidoreductase	*S.* sp. Ru73	92.2%	WP_103830969
4061	dTDP-4-keto-6-deoxy-L-hexose 2,3-reductase	*S. avermitilis*	79.2%	BAA84599.1
4062	dTDP-6-deoxy-L-hexose 3-O-methyltransferase	*S. avermitilis*	85.6%	BAA84598.1
4063	dTDP-4-keto-6-deoxy-L-hexose2,3-dehydratase	*S. avermitilis*	74.5%	BAA84597.1
4064	dTDP-4-keto-6-deoxyhexose 3,5-epimerase	*S. avermitilis*	67.8%	BAA84596.1
4065	dTDP-4-keto-6-deoxy-L-hexose 4-reductase	*S. avermitilis*	53.9%	BAC68658.1
4066	glycosyltransferase	*S. yerevanensis*	93.8%	WP_033324666
4067	Glucose-1-phosphate thymidylyltransferase	*S. avermitilis*	37.1%	BAA84594.1
4068	dTDP-glucose 4,6-dehydratase	*S. avermitilis*	62.4%	BAC68656.1

Figure S1. Maximum-likelihood tree showing the phylogenetic position of strain jys28^T and related taxa based on 16S rRNA gene sequences. Only bootstrap values above 50 % (percentage of 1000 replications) are indicated. Bar, 0.002 nucleotide substitutions per site.

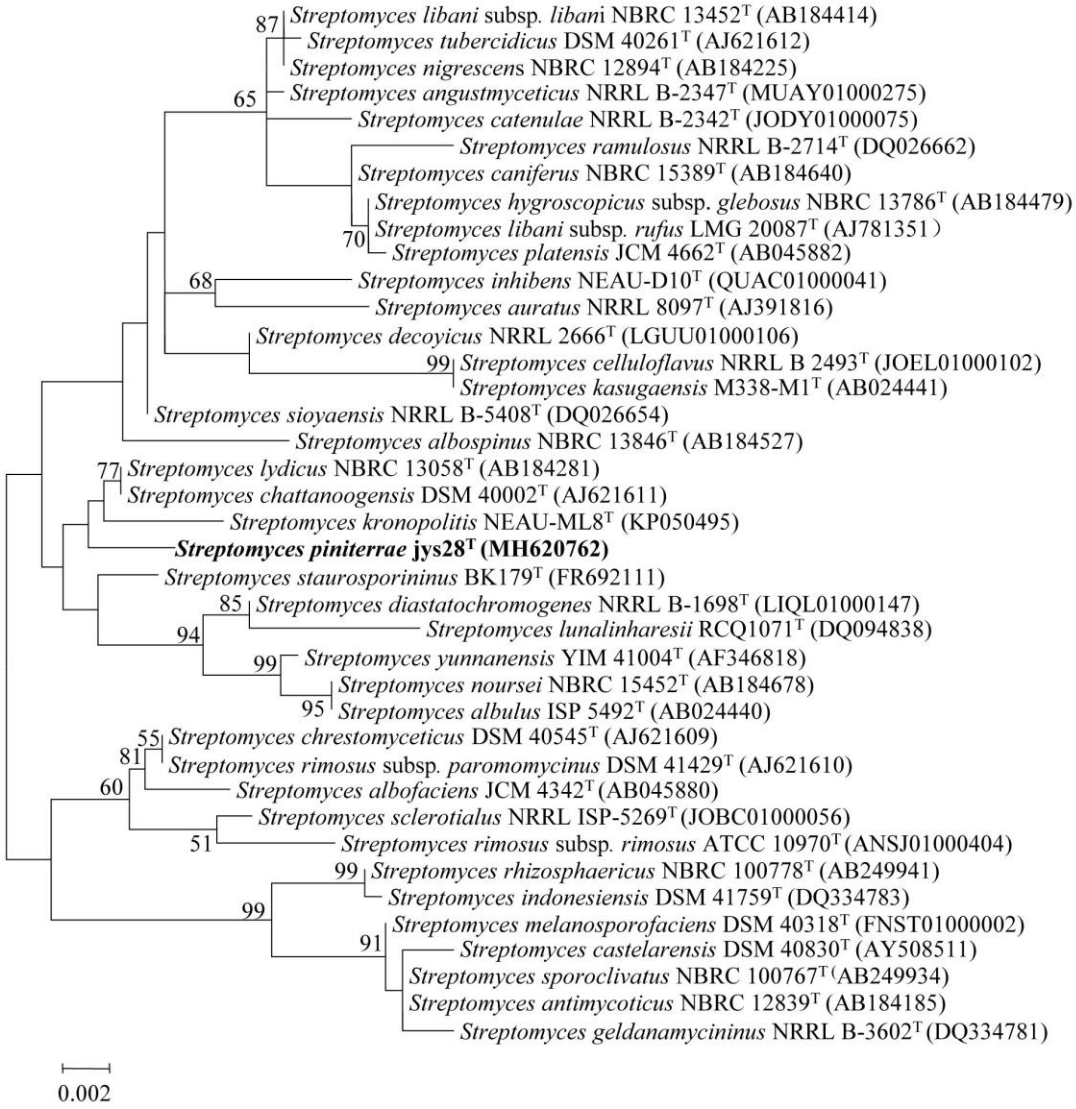

Figure S2. Maximum-likelihood tree based on MLSA analysis of the concatenated partial sequences (2481 bp) from five housekeeping genes (*atp*D, *gyr*B, *rec*A, *rpo*B and *trp*B) of strain jys28^{T} and related taxa. Only bootstrap values above 50 % (percentages of 1000 replications) are indicated. Bar, 0.02 nucleotide substitutions per site.

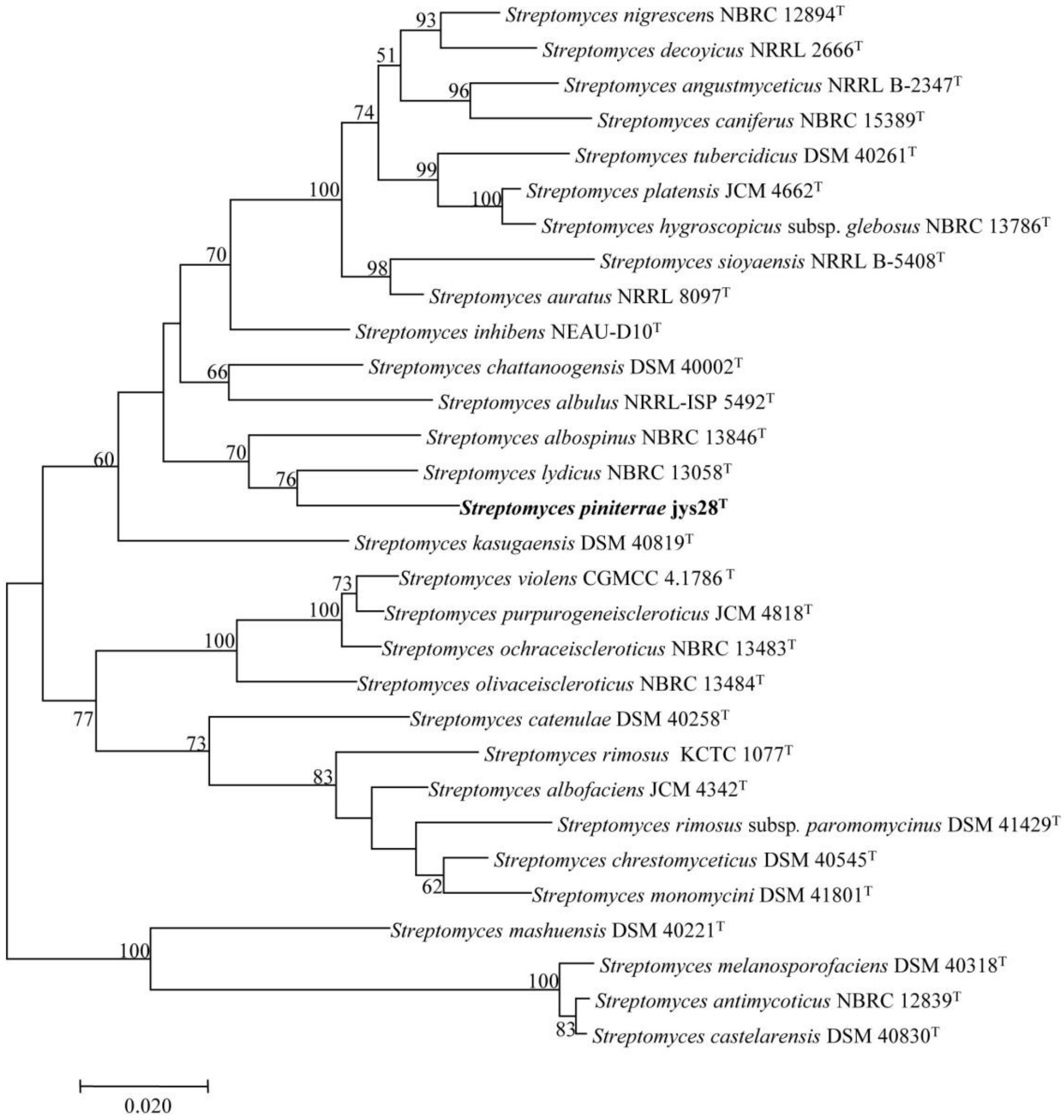

Figure S3. The phospholipids of strain jys28^{T}. a, using molybdenum blue reagent; b, using ninhydrin reagent; c, using anisaldehyde reagent, d, using molybdophosphoric acid reagent. Abbreviations: DPG, diphosphatidylglycerol; PME, phosphatidylmethylethanolamine; PE, phosphatidylethanolamine; PIM, phosphatidylinositol mannoside.

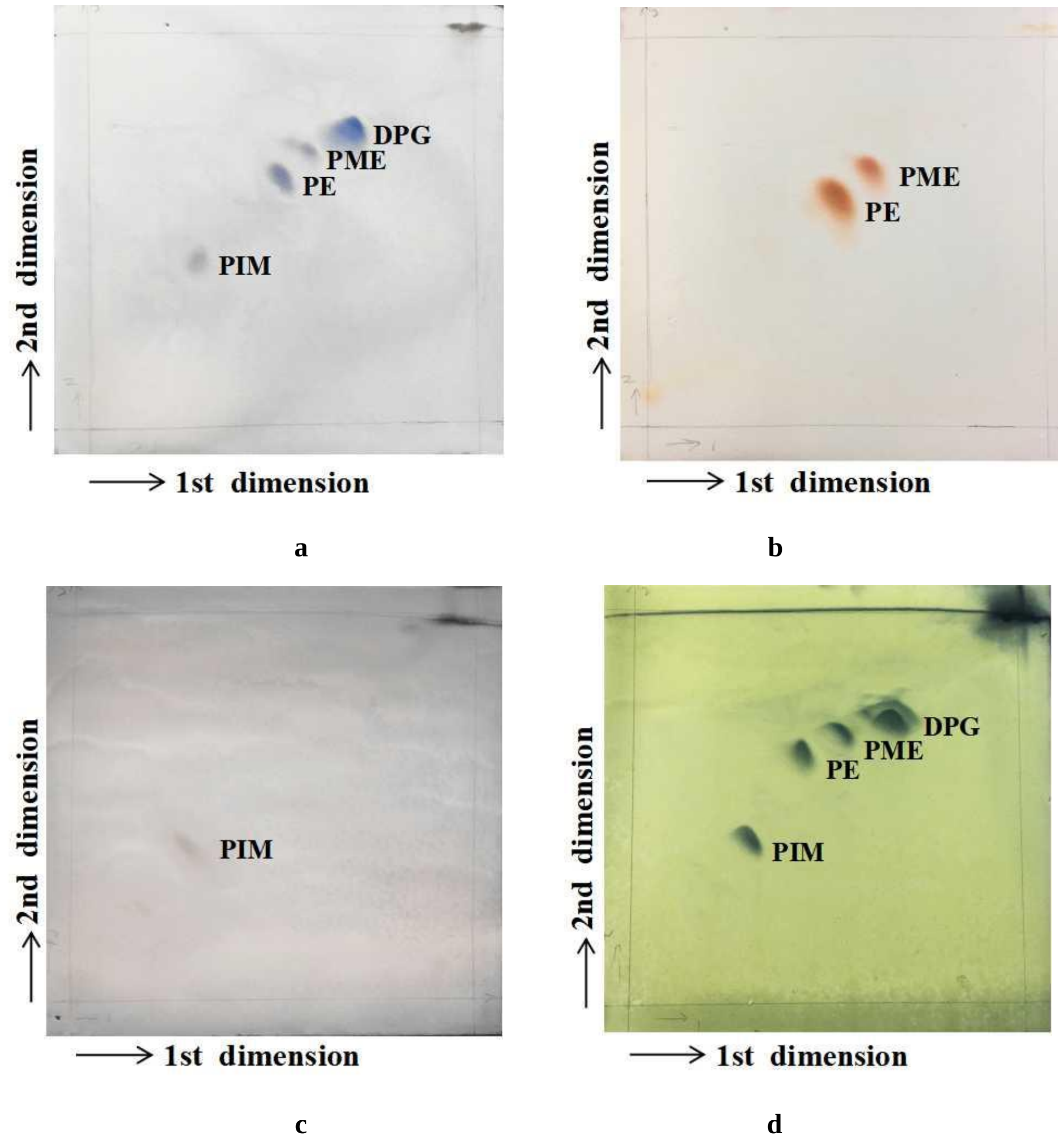

Supplement: Supplementary file 1 [file microorganisms-08-00495-s001.pdf]
